# Meta-analysis on the Effectiveness of ECG Screening for Conditions Related to Sudden Cardiac Death in Young Athletes

**DOI:** 10.1177/00099228231152857

**Published:** 2023-02-16

**Authors:** Nicolas K. Goff, Alexander Hutchinson, Wouter Koek, Deepak Kamat

**Affiliations:** 1 Dell Medical School, The University of Texas at Austin, Austin, TX, USA; 2 Medical University of South Carolina, Charleston, SC, USA; 3 The University of Texas Health Science Center at San Antonio, San Antonio, TX, USA

**Keywords:** cardiology, general pediatrics, adolescent medicine

## Abstract

Controversy exists over the use of electrocardiograms (ECGs) in sports pre-participation screening. We performed a meta-analysis comparing the effectiveness of history and physical examination (H&P) with ECG at detecting both cardiac disease and sudden cardiac death–associated conditions (SCD-AC). Pre-participation studies published from 2015 to 2020 with athletes 10 to 35 years old were included. This yielded 28 011 athletes screened and 124 cardiac diagnoses, 103 of which were SCD-AC. A meta-analysis of log odds ratios (ORs) was conducted using a random-effects model. The ORs for the association between H&P and detecting both cardiac disease and SCD-AC were not statistically significant (OR = 3.4, *P* = .076; OR = 2.9, *P* = .078). The ORs for the association between ECG and detecting both cardiac disease and SCD-AC were statistically significant (60, *P* < .001; 148, *P* < .0001). In conclusion, the odds of detecting both cardiac disease and conditions related to SCD with ECG are greater than with H&P during sports pre-participation screening.

## Introduction

Sudden cardiac death (SCD) in young athletes is a devastating event that may be preventable by effective pre-participation screening; however, there is no universally accepted screening method for the conditions associated with SCD. In Italy, a pre-participation electrocardiogram (ECG) screening program has been in place since 1982, and a previous study by Corrado et al^1^ has shown that it has significantly decreased the incidence of SCD. However, a comparative study by Maron et al has disputed these findings.^2^ The American Heart Association (AHA) and the Association for European Paediatric Cardiology (AEPC) both recommend a screening history and physical examination (H&P) be performed. The AEPC guidelines also recommend a 12-lead ECG be performed before clearing an athlete to play, while the AHA does not. The last meta-analysis on the subject was published in 2015. That same year, an international group of cardiologists came to a consensus on an update to ECG interpretation criteria in athletes.^3^ This was the third such update since 2010, following the publication of the European Society of Cardiology (ESC) criteria in 2010 and the Seattle criteria in 2013, indicating a need for a new meta-analysis to reflect these updates.^4,5^ Therefore, we sought to conduct a meta-analysis of the literature between 2015 and 2020 to compare the value of H&P with that of a 12-lead ECG as a screening tool for detecting cardiac disease.

## Methods

Preferred Reporting Items for Systematic Reviews and Meta-Analyses (PRISMA) guidelines were followed when conducting this meta-analysis. An electronic search of the databases PubMed, MEDLINE, and EMBASE was performed. The search was limited to prospective and retrospective studies published in English between January 1, 2015 and June 30, 2020, when the last search was made. This date range excluded any studies that were included in previous meta-analyses on this topic. We used the search terms [“*pre-participation ECG*” OR “*athlete ECG screening*” OR “*athlete pre-participation”* OR “*athlete ECG*” OR “*pre-participation screening*” OR “*pre-participation screening ECG*”] to identify studies on all databases. We excluded reviews and commentaries. Papers screened for this study were first identified by NKG and AH to ensure that they satisfied inclusion criteria.

Studies included in the meta-analysis fulfilled the following criteria: (1) published between January 1, 2015 and June 30, 2020, (2) published in English, (3) contained athletes between 10 and 35 years of age, (4) reported results of history, physical, and ECG.

The following data were extracted from studies included in the meta-analysis: title of the study, sample size, mean or median age of participants, minimum and maximum ages of the participants, sex and racial/ethnic distribution of participants, country of origin, ECG diagnostic criteria used, number of positive tests for H&P, number of positive tests for ECG, and number of cardiac diagnoses after further evaluation. Four studies were excluded after data extraction had begun because of missing data that were critical to our analysis.

For each study, the association of ECG with cardiac disease and the association of H&P with cardiac disease were quantified as odds ratios. Meta-analysis of log odds ratios using a random-effects model with restricted maximum likelihood estimation was conducted with the MAJOR module in jamovi (https://www.jamovi.org), based on the metafor package in R (Viechtbauer, *Journal of Statistical Software*, 36:3, 2010). The association of both ECG and H&P with cardiac disease was then displayed in forest plots showing 95% confidence intervals.

Finally, a second meta-analysis was run for the association of ECG with conditions associated with SCD. In calculating odds ratios, conditions deemed not to be associated with SCD were not removed completely, but were instead reclassified as “healthy” for the purposes of this analysis. The meta-analysis was calculated in the same manner as the previous one, with the association of both H&P and ECG with conditions associated with SCD displayed as forest plots showing 95% confidence intervals.

The conditions considered to be directly related to increased risk for SCD, and were therefore included in this analysis, were as follows: all cardiomyopathy categories (arrhythmogenic right ventricular cardiomyopathy [ARVC], dilated cardiomyopathy [DCM], hypertrophic cardiomyopathy [HCM], left ventricular noncompaction cardiomyopathy [LVNC], myocarditis), anomalous origin of coronary artery, long QT syndrome, Wolff-Parkinson-White syndrome (WPW), sustained ventricular tachycardia (VT), and SCD unspecified. The structural conditions determined not to be associated with SCD, and therefore removed from this second analysis, included aneurysm with aortic root dilation, aortic coarctation, atrial septum defect, bicuspid aortic valve, congenital coronary atrioventricular fistula, mitral valve prolapse, and right ventricular compression from pectus excavatum. The cardiac rhythm conditions deemed not to be commonly associated with SCD, and therefore excluded from this second analysis, included atrial fibrillation, supraventricular tachycardia, and premature ventricular contraction (PVC) frequent.

A statement of ethics is not required for this study as it based solely on published literature.

## Results

**Figure 1.**
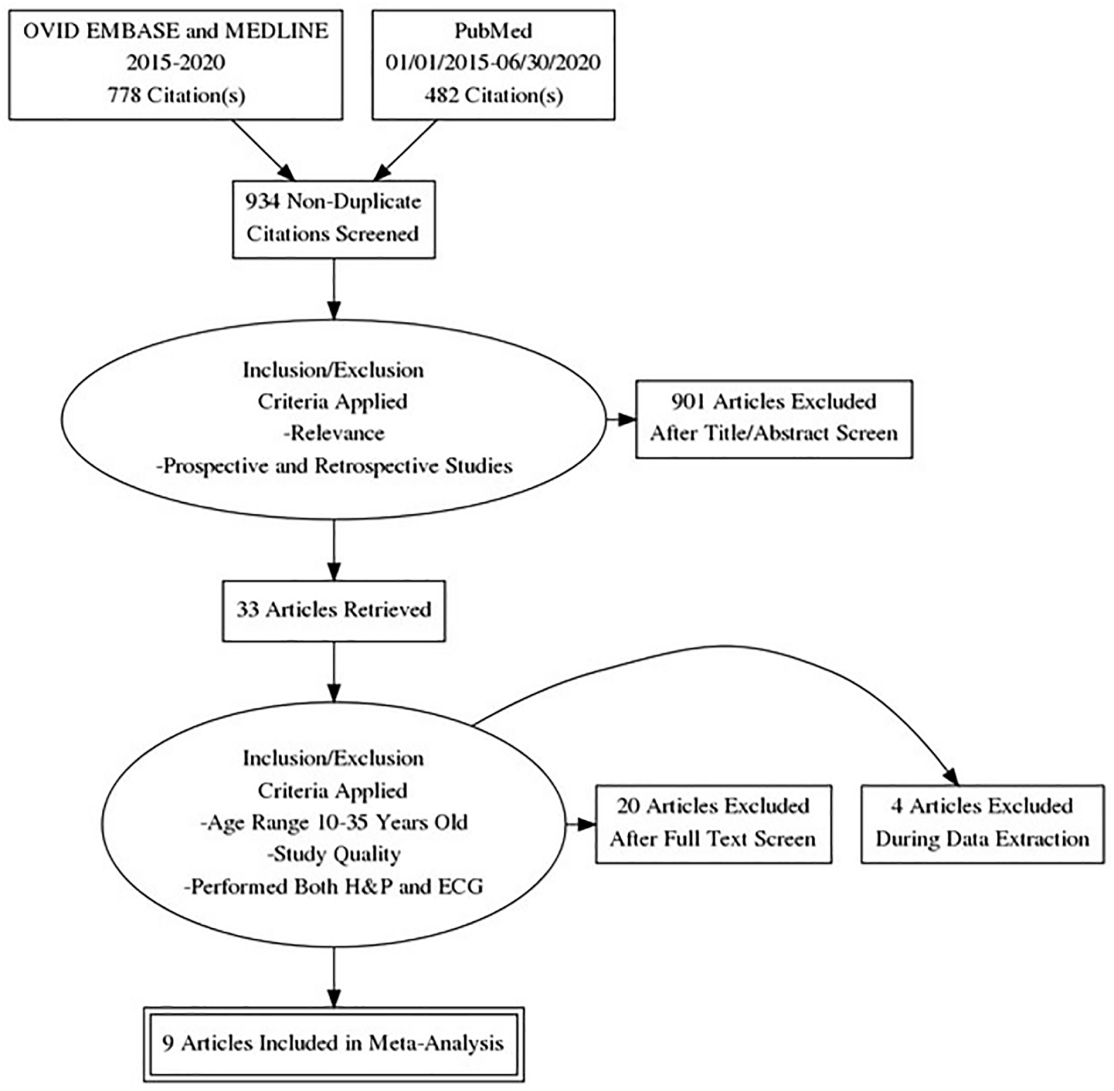
PRISMA flowchart outlining the various databases used and inclusion criteria applied. The flowchart also shows how many studies were included after each round of assessment. Abbreviations: ECG, electrocardiogram; H&P, history and physical examination; PRISMA, Preferred Reporting Items for Systematic Reviews and Meta-Analyses.

Figure 1 is a PRISMA diagram detailing the study selection process. PubMed identified 482 citations and the OVID databases (MEDLINE and EMBASE) identified 778 citations. All searches were downloaded into EndNote 20 and duplicates were removed. After this process, 934 nonduplicate papers were screened for inclusion. After screening the titles and abstracts, 901 publications were excluded, and an additional 20 were excluded after a full review of the papers.

**Figure 2.**
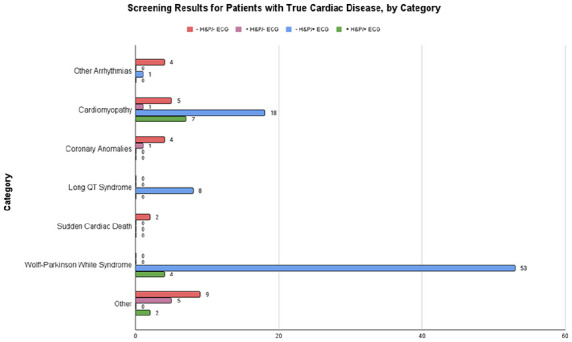
Bar graph showing the results of ECG and H&P for each of the 8 categories of cardiac disease identified in our study population. Abbreviations: ECG, electrocardiogram; H&P, history and physical; SCD, sudden cardiac death.

The characteristics of the studies included in the quantitative analysis are reported in Table 1. Thirteen studies met inclusion criteria, but 4 did not report all metrics necessary and their authors could not be reached for additional data or did not collect the data we required. A total of 28 011 athletes were included in the final analysis, with the study sizes ranging from 516 to 11 168. A total of 21 574 athletes (77%) were identified as male, whereas 12 415 identified as White, 1963 as Black, 430 as Asian/Pacific Islander, and 1160 as other/mixed race. An additional 11 882 athletes did not have their race reported. Three studies were conducted in the United States, 2 in the United Kingdom, and 1 each in Canada, Denmark, Qatar, and Spain. Across all studies, 2857 positive H&P screens and 1125 positive ECG screens were recorded. Of these, 124 new cardiac diagnoses were done. The various cardiac conditions found across all studies as well as broad categories (cardiomyopathies, arrhythmias, etc) can be found in Table 2. Figure 2 shows how each screening method performed in detecting each condition in each category.

**Table 1.** General Characteristics of Studies Included in our Meta-Analysis.

Author	Journal	Year	Country of origin	No. of Athletes screened	Age range, y, (mean) or [median] age	Males, No. (%)
Malhotra et al^6^	*New England Journal of Medicine*	2018	United Kingdom	11 168	15-17 (16.4)	10 581 (95)
Drezner et al^7^	*Journal of the American College of Cardiology*	2015	United States	790	17-25 (18)	444 (56)
McClean et al^8^	*Heart*	2019	Qatar	1304	11-18 (15.1)	1304 (100)
Grazioli et al^9^	*European Journal of Preventative Cardiology*	2017	Spain	1650	12-18 (15.09)	986 (60)
Conway et al^10^	*Clinical Journal of Sports Medicine*	2020	United States	1686	16-25 [18]	993 (59)
Drezner et al^11^	*American Journal of Cardiology*	2016	United States	5258	18-25 (20.1)	2892 (55)
McKinney et al^12^	*Canadian Journal of Cardiology*	2017	Canada	714	12-35	Not specified
Dhutia et al^13^	*Journal of the American College of Cardiology*	2016	United Kingdom	4925	14-35 (19.9)	4068 (83)
Tischer et al^14^	*Scandinavian Journal of Medicine and Science in Sports*	2015	Denmark	516	13-35 (21.58)	306 (59)
Total			7 Countries	28 011	11-35	21 574 (77)

**Table 2.** Conditions Identified From Included Studies and Their Classifications.

Category (Abbr.)	Condition	No. of diagnoses
Arrhythmia, other	Atrial fibrillation	1
Arrhythmia, other	PVC frequent	1
Arrhythmia, other	Supraventricular tachycardia	2
Arrhythmia, other	Sustained ventricular tachycardia*	1
Cardiomyopathy	ARVC*	5
Cardiomyopathy	Dilated cardiomyopathy*	2
Cardiomyopathy	Hypertrophic cardiomyopathy*	19
Cardiomyopathy	Left ventricular noncompaction*	2
Cardiomyopathy	Myocarditis*	3
Coronary anomaly	Anomalous origin of left coronary artery*	1
Coronary anomaly	Anomalous origin of a coronary artery*	3
Coronary anomaly	Congenital coronary AV fistula	1
Long QT	Long QT syndrome*	8
Other	Aneurism with aortic root dilation	1
Other	Aortic coarctation	1
Other	Atrial septum defect	4
Other	Bicuspid aortic valve	7
Other	Mitral valve prolapse	2
Other	RV compression from pectus excavatum	1
Sudden cardiac death, unspecified etiology (SCD)	Sudden cardiac death, unspecified etiology*	2
WPW	Wolff-Parkinson-White syndrome*	57
	Total	124

Abbreviations: PVC, premature ventricular contraction; ARVC, arrhythmogenic right ventricular cardiomyopathy; SCD, sudden cardiac death; WPW, Wolff-Parkinson-White syndrome.

* Conditions associated with sudden cardiac death.

**Figure 3.**
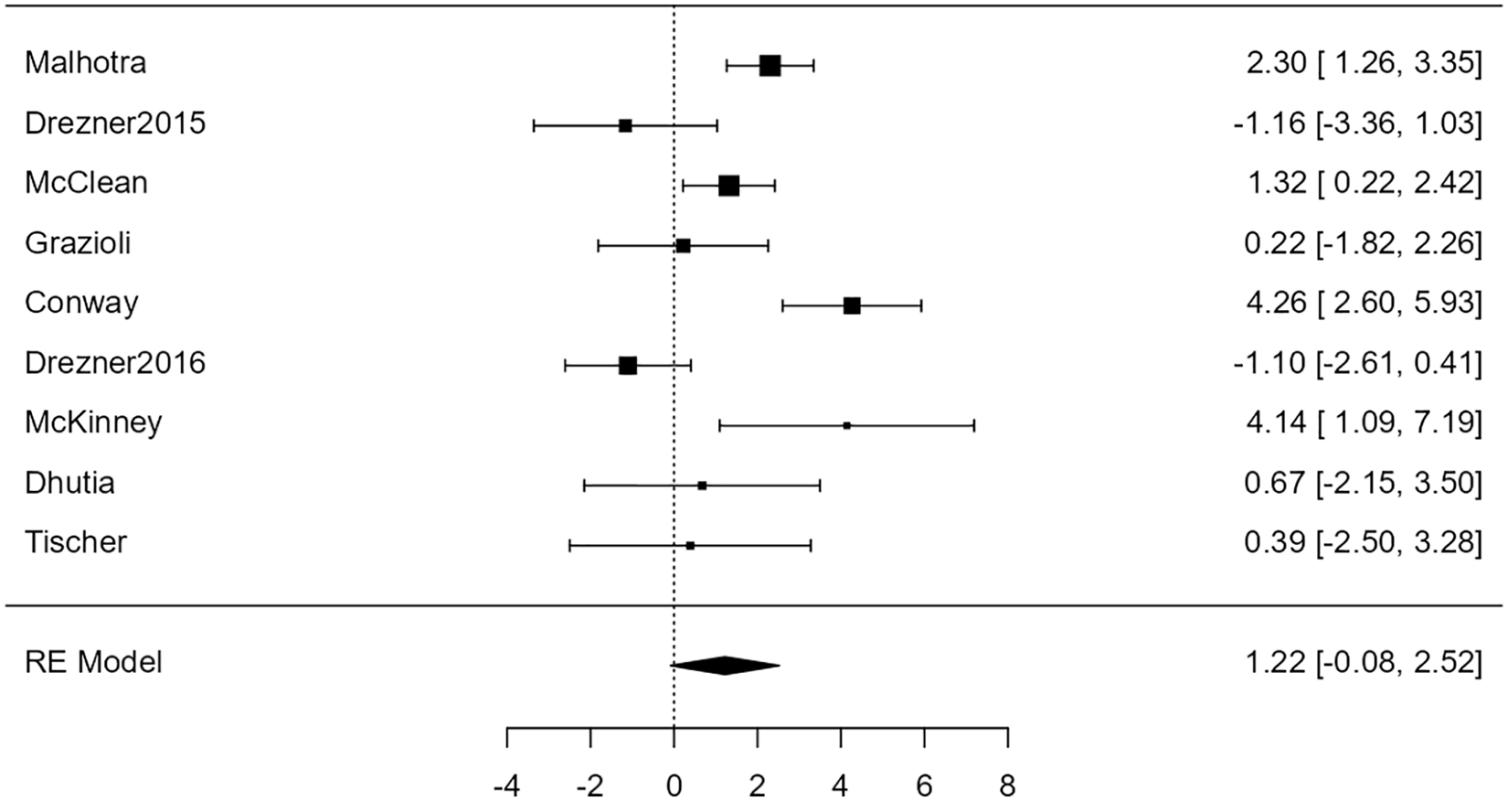
Forest plot showing the results of the association of H&P with cardiac disease. It shows the log odds ratio along with a 95% confidence interval for each of the individual studies included in the meta-analysis as well as an overall estimate based on the random-effects model. These were then back-transformed into odds ratios. Abbreviations: H&P, history and physical examination. RE, random effects.

For the association of H&P with cardiac disease, the random-effects model was applied to the 9 studies, which showed high heterogeneity (*I*^2^ = 79%) yielded a back-transformed odds ratio of 3.4 (95% confidence limits: 0.88-13) that was not statistically significant at the 5% level (*Z* = 1.77, *P* = .076). Figure 3 is a forest plot showing the association of H&P with cardiac disease for each of the 9 studies included in the meta-analysis. These results are expressed as log odds ratios with corresponding 95% confidence intervals, and the overall meta-analysis results are expressed as a log odds ratio based on a random-effects model.

**Figure 4.**
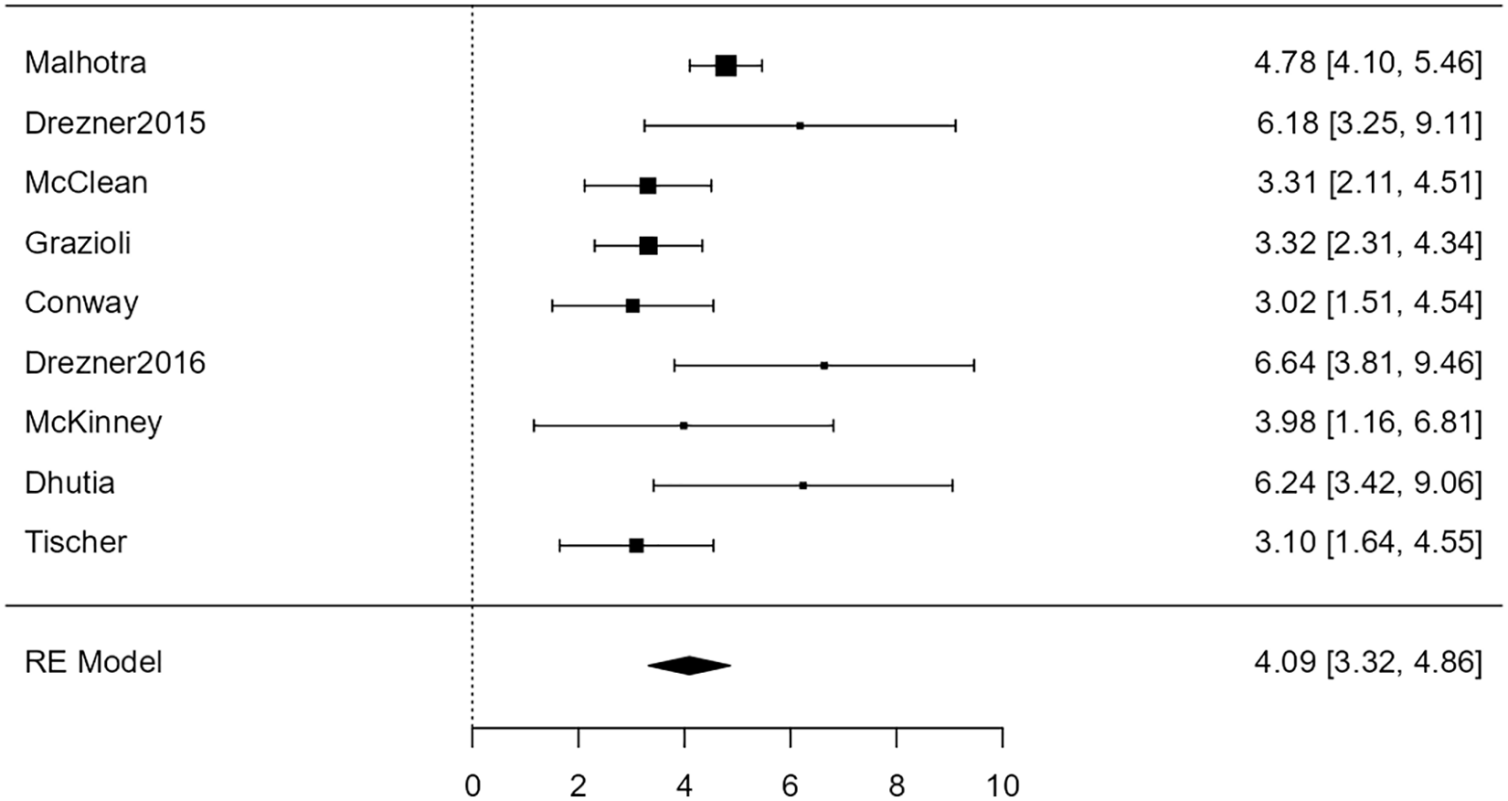
Forest plot showing the results of the association of electrocardiogram with cardiac disease. It shows the log odds ratio along with a 95% confidence interval for each of the individual studies included in the meta-analysis as well as an overall estimate based on the random-effects model. These were then back-transformed into odds ratios. Abbreviation: RE, random effects.

For the association of ECG with cardiac disease, the random-effects model was applied to the 9 studies, which showed moderate heterogeneity (*I*^2^ = 58%) yielded a statistically significant (*Z* = 9.61, *P* < .001) back-transformed odds ratio of 60 (95% confidence limits: 26-137). Figure 4 is a forest plot showing the association between ECG and cardiac disease.

**Figure 5.**
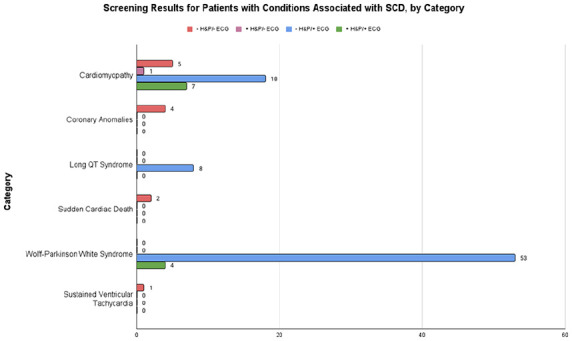
Bar graph showing the results of ECG and H&P for each of the 6 categories of conditions associated with SCD. Abbreviations: ECG, electrocardiogram; H&P, history and physical; SCD, sudden cardiac death.

After this first round of analysis, a second meta-analysis was performed for the association of H&P and ECG with conditions associated with SCD. Multiple cardiologists were consulted in determining which conditions to remove. In the end, 103 cardiac diagnoses associated with SCD were made across all 9 studies. Figure 5 shows the screening method used to detect each type of condition.

**Figure 6.**
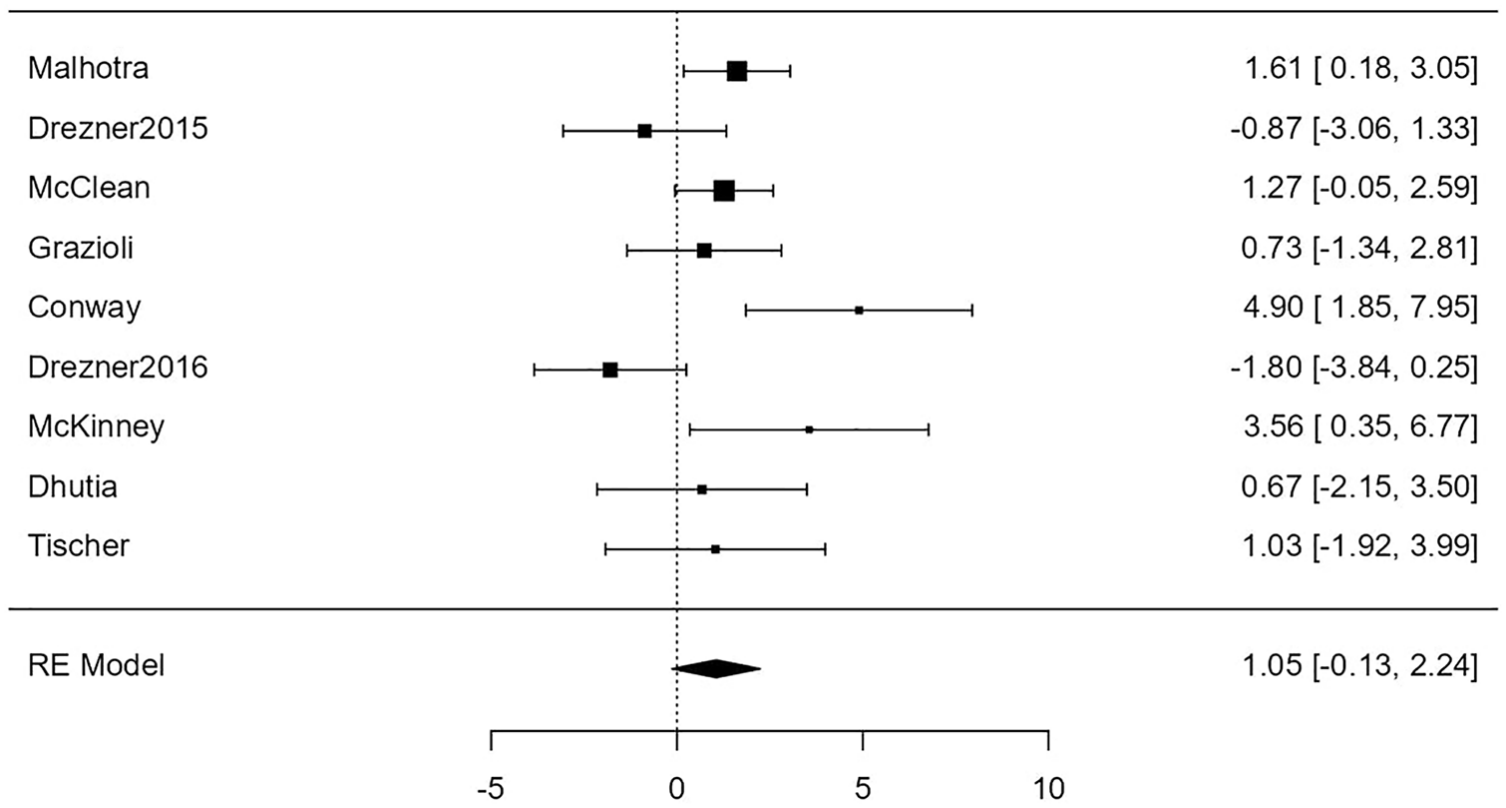
Forest plot showing the results of the association of H&P with conditions associated with sudden cardiac death. It shows the log odds ratio along with a 95% confidence interval for each of the individual studies included in the meta-analysis as well as an overall estimate based on the random-effects model. These were then back-transformed into odds ratios. Abbreviations: H&P, history and physical examination. RE, random effects.

For the association of H&P with conditions associated with SCD, the random-effects model was applied to the 9 studies, which showed high heterogeneity (*I*^2^ = 63%). It yielded a back-transformed odds ratio of 2.9 (95% confidence limits: 0.88-9.4) that was not statistically significant at the 5 percent level (*Z* = 1.74, *P* = .082). Neither the rank correlation nor the regression test indicated any funnel plot asymmetry (*P* = .26 and *P* = .30, respectively). Figure 6 shows the association of H&P with conditions associated with SCD for each of the 9 studies as well as the overall meta-analysis as a forest plot.

**Figure 7.**
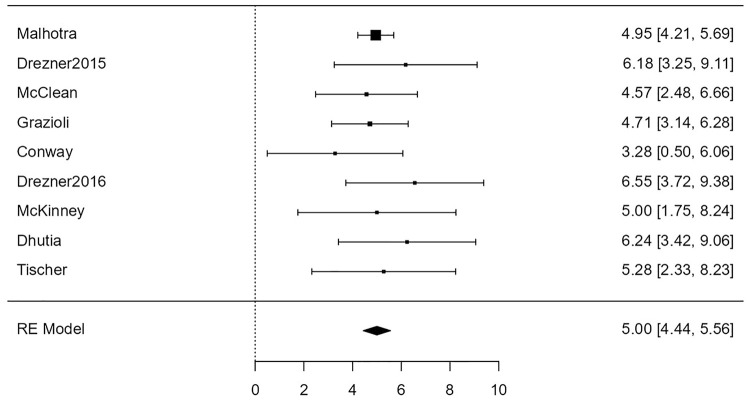
Forest plot showing the results of the association of electrocardiogram with conditions associated with sudden cardiac death. It shows the log odds ratio along with a 95% confidence interval for each of the individual studies included in the meta-analysis as well as an overall estimate based on the random-effects model. These were then back-transformed into odds ratios. Abbreviation: RE, random effects.

For the association of ECG with conditions associated with SCD, the random-effects model was applied to the 9 studies, which showed no heterogeneity (*I*^2^ = 0%), yielding a statistically significant (*Z* = 17.44, *P* < .0001) back-transformed odds ratio of 148 (95% confidence limits: 84-260). Neither the rank correlation nor the regression test indicated any funnel plot asymmetry (*P* = .61 and *P* = .60, respectively). Figure 7 shows this association between ECG and conditions associated with SCD as a forest plot.

## Discussion

Since the publication of the Corrado study in 1998 detailing the implementation of a screening program in the Veneto region of Italy,^1^ there has been a lively debate among cardiologists over the inclusion of a 12-lead ECG in pre-participation screening for young athletes. A subsequent study published by Corrado was not a controlled trial designed to compare screening with an ECG with screening without an ECG, leaving the impact of the results up for interpretation.^15^ There are strong arguments on both sides, with the group in favor of universal ECG screening often citing the 2006 study of Corrado et al^15^ showing a consistent drop in SCD since the beginning of the screening to 2004. Some in-roads have been made in studying the feasibility of including a universal ECG for pre-participation screening. The practicality of such a program has long been a consistent concern for group opposing using ECG for screening, especially in the United States. Marek et al performed ECGs on 32 561 high school students in Chicago and found that such a program was practical—even when performed by volunteers under cardiologist supervision—with only 0.81% of ECGs having to be redone due to technical issues. This study provided the ECGs at no cost to the students; however, they did estimate the cost of performing and reading ranged from $13.17 to $18.19.^16^ The Association of European Paediatric Cardiology’s (AEPC) guidelines for pre-participation screening were updated in 2017 to include a 12-lead ECG in their screening programs. The AEPC is the first pediatric cardiology organization to do so.^17^ In addition, many professional sports leagues, the International Olympic Committee and the ESC, have published similar recommendations.

Current AHA guidelines, on the contrary, do not recommend the use of a 12-lead ECG in pre-participation screening for young athletes.^18^ A 2017 survey of 205 pediatricians showed that 92% of those who performed pre-participation exams did not include an ECG because of those guidelines.^19^ Furthermore, 63% of those pediatricians felt that they could not independently interpret an ECG, and many of those who did not currently perform ECG screening were concerned about out-of-pocket costs for patients and unnecessary restrictions on sport participation.^19^ Another argument against mandating the inclusion of a 12-lead ECG in pre-participation screening is that the observed drops in SCD rates after implementing a screening program are not statistically significant and are due to normal fluctuations. For example, Israel passed a law mandating universal ECG screening for athletes in 1997, which was considered successful because SCD rates dropped the following year. In a 2011 study, however, Steinvil et al^20^ shows that the SCD rate continued to fluctuate throughout the entire study period of 1985 to 2008, and that the time period with the lowest incidence of SCD was 1988 to 1989, long before the implementation of the screening program.

According to a review by Corrado and Zorzi,^21^ the most common causes of SCD in young athletes are due to structural and/or electrical abnormalities. Common structural causes include HCM, arrhythmogenic ventricular cardiomyopathy, coronary anomalies, myocarditis, and valve diseases. Electrical causes include long QT syndrome, VT, WPW, and Brugada syndrome.^21^ Estimations of the incidence of SCD vary, from 0.11 per 100 000 athlete-years to 4.4 per 100 000 athlete-years.^22,23^ A comprehensive review of the literature published in 2014 placed the incidence of SCD at 2 per 100 000 athlete-years.^24^

The previous meta-analysis on the subject, performed by Harmon et al^25^ in 2015, included papers published between 1996 and 2014. This study concluded that ECG was a more effective screening tool than H&P for screening conditions associated with SCD and recommended that it be implemented as the primary method for screening athletes for conditions associated with SCD.^25^ The 2014 cutoff, however, excluded studies performed using the updated 2015 international criteria, as well as studies which used the Seattle criteria published in 2013.

We sought to compare the effectiveness of H&P with the effectiveness of a 12-lead ECG in detecting cardiac disease. This includes conditions associated with SCD, such as HCM or WPW, as well as unrelated conditions, such as mitral valve prolapse. Every condition found by a screening program was categorized, as shown in Table 2. Arrhythmogenic right ventricular cardiomyopathy—now commonly known as arrhythmogenic ventricular cardiomyopathy—DCM, HCM, myocarditis, and LVNC were categorized as cardiomyopathies. We included congenital coronary atrioventricular fistula, anomalous origin of the left coronary artery and other, unspecified coronary artery anomalies under the category of coronary anomalies. Four conditions, WPW, long QT syndrome, and SCD with unspecified etiology, were listed as individual categories. “Arrhythmias, other” included atrial fibrillation, supraventricular tachycardia, sustained VT, and frequent PVCs. Finally, there were multiple structural conditions listed as “other,” including an aneurysm with aortic root dilation, aortic coarctation, atrial septum defect, bicuspid aortic valve, mitral valve prolapse, and pectus excavatum with ventricular compression. Three athletes with 2 diagnoses—all from the Conway study—were removed because their condition was not considered pathological. The first was one athlete with dextrocardia. He already knew of his condition, but failed to mention it until the ECG screen returned abnormal results.^10^ Two diagnoses of patent foramen ovale were also removed.

Figure 2 shows the performance of screening methods in detecting each category. Some conditions, such as long QT syndrome, were only detected by ECG. WPW was detected only by ECG as well, except for 4 cases that also had positive H&P. Of these 4 positive H&Ps, 2 were due to incidental murmurs found during the physical examination, 1 was due to a family history of SCD, and 1 was unspecified. The conditions listed under “arrhythmias, other” were not detected by any screening technique considered by this meta-analysis. All cases of SCD with unspecified etiology were only found after the death of the patient. Of the 2 cases of supraventricular tachycardia, 1 was detected by ECG and the other was found by a stress test and an electrophysiological study. Only one coronary anomaly, a congenital coronary atrioventricular fistula, was detected by H&P. The rest were identified by echocardiography, which was not included as a screening modality in this meta-analysis. Cardiomyopathies were more commonly detected by ECG or both H&P and ECG (18 and 7 of 31 total cases, respectively). Among the 19 “other” conditions, 6 cases were detected via H&P alone, 2 via ECG alone, 2 via ECG and H&P, and 9 via methods not included in this meta-analysis.

We found that the odds of detecting cardiac disease with a 12-lead ECG were overall statistically significant (*P* < .001). In addition, 8 of the 9 studies included showing statistically significant odds ratios for the ECG (Figure 4). On the contrary, the odds of detecting cardiac disease with H&P were not statistically significant (*P* = .076) (Figure 3). These results suggest that a 12-lead ECG is a more effective tool than H&P for screening cardiac disease associated with SCD.

Four studies did show statistically significant odds of detecting cardiac disease using H&P alone. The 95% confidence intervals of the log odds ratios for these studies can be found in Figure 3. The most likely explanation for this deviation from our observation is that our meta-analysis includes a much larger sample size than these individual studies. However, the largest study, included in our meta-analysis, performed by Malhotra et al^6^ did show statistically significant odds of detecting cardiac disease by H&P alone.

We found that the association between H&P and conditions associated with SCD was similar to that between H&P and cardiac disease. The odds ratio decreased slightly, from 3.4 to 2.9, but the overall effect did not change, with both associations being not statistically significant at the 5% level (*P* = .076 and *P* = .078, respectively).

There was a major difference, however, between the association of ECG and cardiac disease and the association of ECG and conditions associated with SCD. The odds ratio increased from 60 for the former to 148 for the latter. Although both were statistically significant with a *P* value well below .001, this increase can only serve as further evidence of the effectiveness of the ECG in pre-participation screening.

## Limitations

Our study does have some limitations. First, we were unable to perform an analysis of the effectiveness of ECG along with H&P as a screening tool because of the way data were reported in some of the papers. Second, our study only focuses on the screening ability of the 2 tools, not their feasibility. Performing 12-lead ECGs consumes resources and time, and the incidences of conditions associated with SCD are very low. Another consideration is that the ECG is an excellent tool to detect electrical abnormalities, but is not diagnostic for structural abnormalities, and is not a reliable tool to identify coronary anomalies, which are also associated with SCD.^21^ In addition, we did not have information regarding who performed the H&Ps, as there is the potential for large variations of training level/experience of the medical providers performing the H&Ps. Finally, the studies included in our meta-analysis did not use the same ECG criteria for screening. Of the 9 studies included in this meta-analysis, 7 used the Seattle criteria.^5^ The Conway and Tischer studies used a combination of the Seattle and other criteria, but for different reasons. The Conway study compared various criteria, but the screening data reported in this meta-analysis were collected using the Seattle criteria.^10^ The Tischer study used the 2010 ESC criteria for screening, but some analyses were performed later using the Seattle criteria.^4,14^ Finally, the findings of this study are somewhat limited to the population of white men. The patient population of this meta-analysis was 77% male and majority white, though racial data were unavailable for a large proportion of the study population.

Future research on the subject should be focused on large, prospective studies on young and diverse populations around the world. These large screening programs, such as the one used by the English Football Association described by the Malhotra study, are the best way to provide strong evidence for effective screening tools.^6^

Further research should also be done to determine the costs in time, finances, and resources associated with universal ECG screening program for young athletes. Especially, in the United States, where a pre-participation exam is often charged to the athlete or their family, this information is necessary to determine its feasibility and affordability. Recent research has demonstrated a decreasing cost of universal screening. For example, Assanelli et al studied the cost-effectiveness of universal ECG screening in France, Germany, Greece, and Algeria. The average cost of screening per athlete was $60.50, and the cost-effectiveness ratio was estimated to be $4071 per life-year saved in Europe and $582 per life-year saved in Algeria. These estimates were based on costs for ECG screening ranging between $14.73 and $31.11, with costs increasing with further workup.^26^ A second study, performed on a cohort of 891 Swiss pediatric athletes, provided an average cost of $122 per athlete screened.^27^

As technology improves and 12-lead ECGs become less expensive to perform and easier to read, the feasibility of universal ECG screening will increase. One of the most exciting advances is in the field of artificial intelligence with its ability to identify cardiac anomalies at a rapid rate with accuracy. Bos et al^28^ showed that a 10-second 12-lead artificial intelligence-read ECG allowed for the identification of concealed long QT syndrome with improved sensitivity and specificity compared with standard interpretation criteria. Furthermore, a 2019 study on a large cohort of patients (44 959 training and 52 870 testing) showed sensitivity, specificity, and accuracy values of 86.3%, 85.7%, and 85.7%, respectively, when screening for ventricular dysfunction.^29^ Although the technology is still in its infancy, these studies show that artificial intelligence may provide a cost-effective solution to universal ECGs in the near future.

## Conclusion

By conducting a meta-analysis with a large number of patients, we have shown that modern ECG criteria improve the odds of detecting cardiac disease in young athletes as compared with history and physical examinations. Our data show that the odds of detecting cardiac disease with a pre-participation ECG are statistically significant (odds ratio of 60, *P* < .001) and higher than the odds of detecting cardiac disease with H&P (odds ratio of 3.4, *P* = .076). We also found that the odds of detecting conditions associated with SCD with a pre-participation ECG are statistically significant (odds ratio of 159, *P* < .001) and higher than the odds of detecting conditions associated with SCD with H&P (odds ratio of 2.9, *P* = .078). We conclude that using a 12-lead ECG as a screening tool improves the odds of identifying cardiac disease and SCD in young athletes.

## Author Contributions

NKG created the search strategy, completed the search, performed data extraction, prepared the figures, and wrote the manuscript. AH was responsible for the conceptualization of the study, data extraction, and editing of the manuscript. WK was responsible for statistical analysis, figure preparation, and writing of the manuscript. DK supervised the project and edited the manuscript.
